# Foreword

**DOI:** 10.1038/sj.bjc.6605267

**Published:** 2009-09-15

**Authors:** A L Jones, R Leonard

**Affiliations:** 1 University College Hospital, 250 Euston Road, London NW1 2PG, UK; 2 Charing Cross Hospital, Fulham Palace Road, London W6 8RF, UK

Chemotherapy is a relatively recent discipline, having its origins in the 1970s, but its success can already be seen in the United Kingdom and across the world. The initial advances, in terms of effective disease management and improved outcomes, were seen in haematological malignancies, and were closely followed by improvements for patients with breast cancer and other solid tumours.

In this supplement, Verrill highlights the increasing use of intensive adjuvant regimens for breast cancer, and the increased rates of febrile neutropenia (FN) associated with these regimens. Development of FN can lead to morbidity, and indeed mortality, for individual patients, and direct costs for the NHS, related to hospital admission and treatment.

Yet neutropenia is often avoidable. One approach, if a patient develops an episode of FN during chemotherapy, is dose reduction during subsequent cycles. However, as Leonard has pointed out (Leonard *et al*, 2003), compromising the dose of chemotherapy in adjuvant treatment of breast cancer may also compromise efficacy.

The National Confidential Enquiry into Patient Outcome and Death (NCEPOD) report into deaths within 30 days of chemotherapy reported that FN was a significant cause of iatrogenic death, and that the standards of care for patients receiving chemotherapy left considerable room for improvement (NCEPOD, 2008). These findings were taken up by the National Chemotherapy Advisory Group (NCAG), which made recommendations for the management of chemotherapy-induced FN with the aim of improving safety and outcome (NCAG, 2009).

These recommendations are extremely important, but can we do more to prevent FN? The use of haematopoietic growth factors will reduce the incidence of both asymptomatic neutropenia and FN (Aapro *et al*, 2006; Smith *et al*, 2006), but it is not practical or cost-effective to provide primary prophylaxis for all patients undergoing chemotherapy. As part of the European Union, maybe it is time for the United Kingdom to consider an approach based on the European guidelines for FN prevention (Aapro *et al*, 2006). Under these guidelines, patients undergoing chemotherapy are triaged for consideration of primary prophylaxis with growth factors according to the intensity of the regimen and patient-specific risk factors.

In an era when we have the ability to cure more patients and prolong high-quality life for those we cannot cure, it beholds us to make sure that our treatment does not create unnecessary morbidity and mortality.

The articles in this supplement consider the burdens imposed by FN, and the strategies available for preventing as well as providing optimum management for this common side effect of many chemotherapy regimens.
